# Fully Atomistic Molecular Dynamics Simulation of Ice Nucleation Near an Antifreeze Protein

**DOI:** 10.1021/jacs.4c15210

**Published:** 2025-01-23

**Authors:** Yue Zhang, Ning Wei, Liwen Li, Yuan Liu, Changxiong Huang, Zhen Li, Yujie Huang, Dengsong Zhang, Joseph S. Francisco, Junhua Zhao, Chunlei Wang, Xiao Cheng Zeng

**Affiliations:** † Jiangsu Key Laboratory of Advanced Food Manufacturing Equipment and Technology, Jiangnan University, Wuxi 214122, China; ‡ School of Petroleum Engineering, China University of Petroleum (East China), Qingdao 266580, China; § Department of Materials Science and Engineering, City University of Hong Kong, Hong Kong, Kowloon 999077, China; ∥ School of Chemical Engineering and Technology, Sun Yat-Sen University, Zhuhai 519082, China; ⊥ International Joint Laboratory of Catalytic Chemistry, Innovation Institute of Carbon Neutrality, College of Sciences, Shanghai University, Shanghai 200444, China; # Department of Earth and Environmental Science, University of Pennsylvania, Philadelphia, Pennsylvania 19104, United States

## Abstract

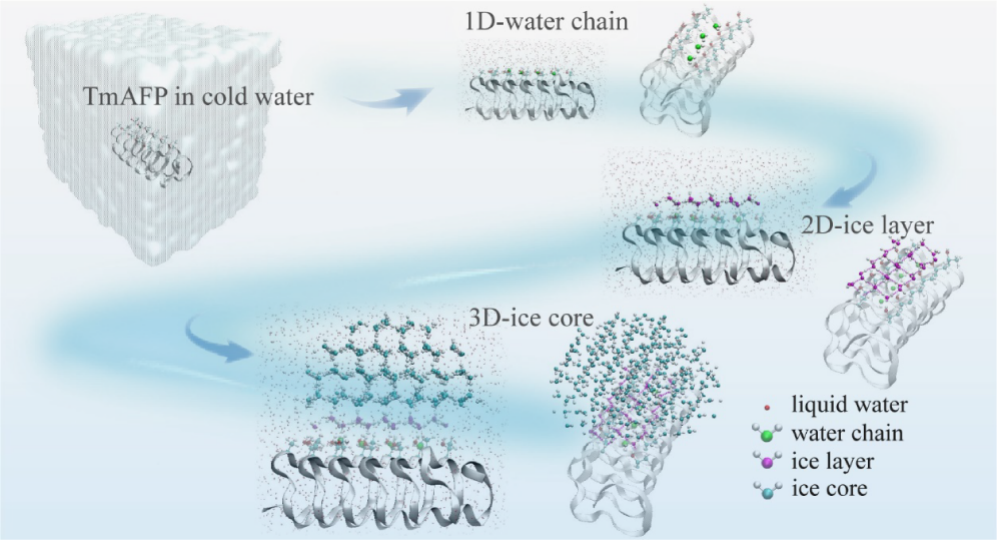

Heterogeneous ice nucleation is a widespread phenomenon in nature. Despite extensive research on ice nucleation near biological antifreeze proteins, a probe for ice nucleation and growth processes at the atomic level is still lacking. Herein, we present simulation evidence of the heterogeneous ice nucleation process on the ice-binding surface (IBS) of the *Tenebrio molitor* antifreeze protein (TmAFP). Our all-atomistic molecular dynamics simulations reveal detailed steps toward precritical nucleus formation from one-dimensional (1D) channel water to a 2D ice nanolayer and, finally, a 3D ice nucleus. Compared with homogeneous ice nucleation under the same supercooling conditions, the IBS of TmAFP can markedly reduce the critical size of the ice embryo and lower the nucleation free energy barrier, thereby favoring ice nucleation. Additionally, through artificial mutation of selected functional groups on the IBS, we gain deeper insights into how the specific functional groups of the IBS affect ice nucleation. We highlight that the carbonyl groups in the backbone play a crucial role by providing fixed locations for channel water. This function is essential for ensuring alignment between the 2D ice nanolayer and the ice lattice structure.

## Introduction

Heterogeneous ice nucleation is prevalent in subfreezing environments because of the ubiquitous presence of surfaces on Earth.^1−4^ For adaptation to cold weather environments, nature has endowed diverse living species with antifreeze proteins (AFPs), thereby enabling them to survive under subfreezing conditions.^5 −11^ Notably, insect AFPs are known to be more potent than fish AFPs at depressing the freezing point of solutions.^12^ Currently, the mechanism of AFPs at the mesoscopic level, namely, the adsorption-inhibition mechanism,^13−15^ is largely understood. Previous studies have shown that AFPs can adhere to the surface of ice crystals through their ice-binding surface.^14,16−19^ This adhesion results in changes in the curvature of the ice surface between adjacent AFPs due to the Kelvin effect,^14,20,21^ effectively lowering the freezing point of ice. Additionally, recent experiments have indicated a “Janus effect” of AFPs, wherein the ice-binding surface can promote ice nucleation in supercooled solutions.^22−24^ Hyperactive AFPs have been reported to bind to ice or facilitate ice nucleation through anchored clathrate or ice-like motifs.^16^ Importantly, the structure of AFPs is very similar to that of bacterial ice-nucleating proteins (INPs). Notably, heterogeneous ice nucleation behavior on the surface of INPs has been investigated via molecular dynamics (MD) simulations, which revealed that, for example, the freezing temperature is strongly affected by the size and aggregation of INPs.^22^ However, for most MD simulations reported in the literature, the coarse-gained mW water model was used.^24−28^ Thus, atomistic details of the ice nucleation dynamics on INPs are still lacking. An MD simulation with a fully atomistic water model would provide more accurate atomistic insight into how the hydrogen-bonding interactions between water molecules and proteins affect the ice nucleation dynamics. Additionally, the atomistic-level understanding of the mechanism underlying the formation of ice-like motifs on the ice-binding surface (IBS) and the specific structural characteristics of the IBS is still incomplete.

Ice nucleation is a critical step in the water freezing process.^26,29−33^ As explained by classical nucleation theory (CNT), the critical nucleation step involves the formation of a critical ice nucleus through overcoming of the free energy barrier via thermal fluctuations.^34−36^ A recent experiment demonstrated a temperature dependence of the critical ice nucleus size.^37^ Heterogenous ice nucleation on carbon supports can also be explained by CNT.^36^ Conventional wisdom suggests that effective ice-nucleating surfaces tend to form strong chemical bonds with water, inducing ice-like order through specific chemical interactions and thus promoting ice formation.^38−41^ However, determination of the specific features that make certain surfaces (such as the IBS of AFPs) more conducive to ice nucleation requires a deeper understanding of heterogeneous ice nucleation at the atomistic level. Therefore, there is a pressing need to simulate spontaneous ice nucleation with fully atomistic models in real time so that mechanistic studies on how hyperactive AFPs influence ice nucleation can be achieved.

In this study, we performed microsecond-scale MD simulations to obtain, for the first time, a dynamic trajectory of heterogeneous ice nucleation and growth on the IBS of the *Tenebrio molitor* AFP (TmAFP) using an all-atom water model. Our simulations reveal an intriguing phenomenon in which the vicinal ice nucleus on TmAFP evolves from a one-dimensional (1D) water chain to a 2D ice nanolayer and then to a 3D ice embryo. More specifically, during ice nucleation, the inverted groove on the IBS anchors a 1D chain of channel water (CW), leading to the formation of an ordered 2D ice-like layer on the IBS. This ice-like layer subsequently promotes the formation of critical ice embryos, enabling rapid growth into 3D crystallites. Compared with the homogeneous nucleation counterpart, the IBS of TmAFP significantly reduces the critical size of the ice nucleus, as well as the nucleation free energy barrier, and facilitates heterogeneous ice nucleation. Finally, selected artificial mutation of TmAFP allows us to gain an atomistic-level understanding of how each specific functional group among the methyl, hydroxyl, and carboxyl groups contributes to the promotion of heterogeneous ice nucleation. The obtained atomistic-level insights will be valuable for the future design and synthesis of biomimetic surfaces to achieve more effective regulation of ice nucleation processes.

## Results and Discussion

### Freezing Behavior of Vicinal Water Induced by TmAFP Under Cryogenic Conditions

**Figure 1:**
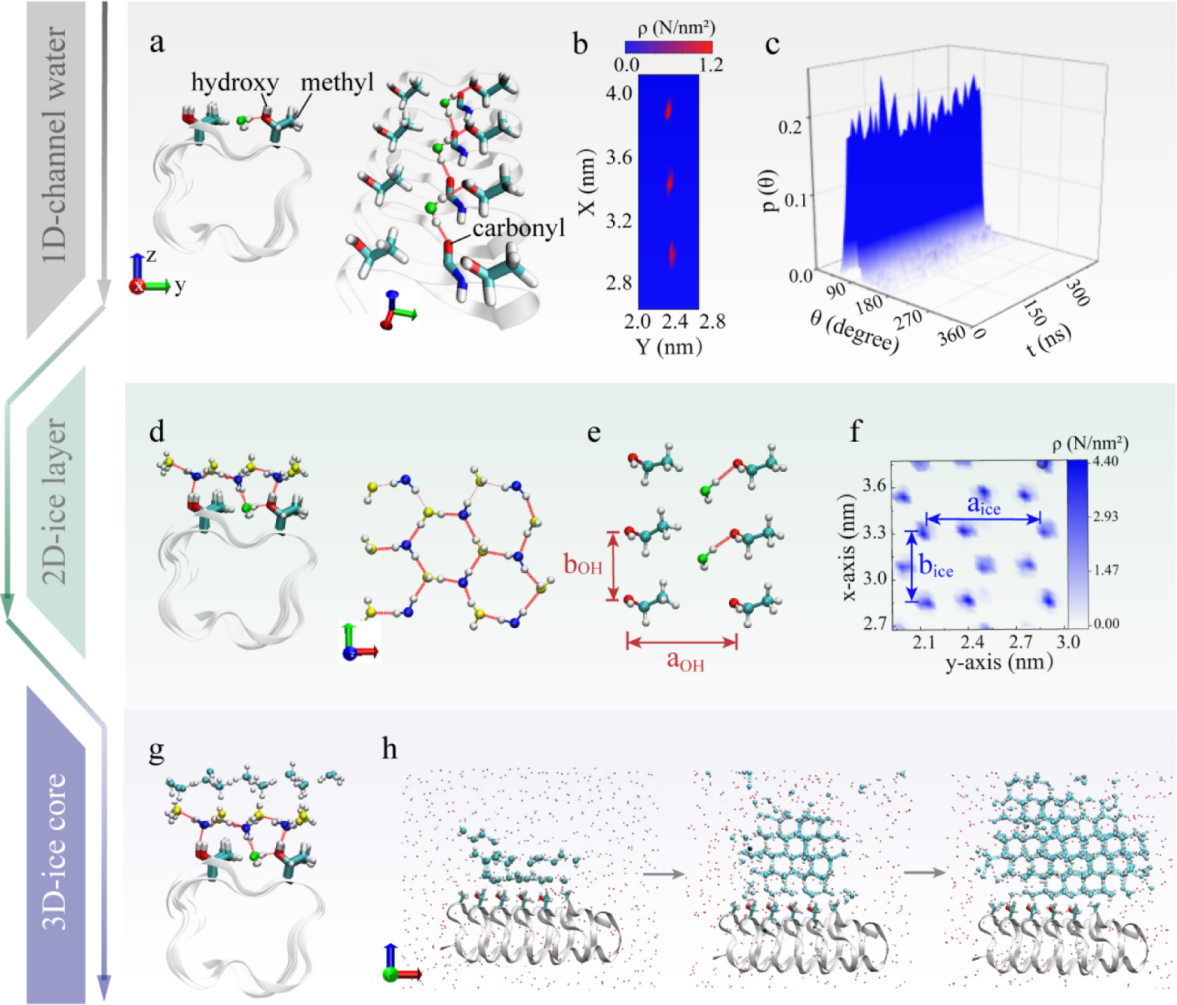
MD simulation evidence of ice nucleation on the IBS of TmAFP. (a) Side and top views of CW anchored within an inverted groove between threonine residues, where the CW forms hydrogen bonds with both the hydroxyl groups on threonine and the carbonyl groups on cysteine. The central backbone of the protein is depicted using a silver cartoon representation, while the functional groups on the IBS are highlighted with colorful sticks. Hydrogen bonds are denoted by red dotted lines. (b) Density distribution of CW in the xy plane over the microsecond simulation. (c) Probability distribution of the dipoles of CW versus the simulation time. Here, θ is defined as the angle between the dipole of the water molecule and the *x*-axis. (d) Side and top views of a snapshot of an ordered ice-like layer on the IBS, with the ice-like layer in contact with the protein being represented by spheres in shades of blue–white and yellow–white. (e) The pattern of aligned hydroxyl groups (at the ice-binding site of TmAFP) resembles the ice lattice, as evidenced from (f) the density distribution of the ice-like layer parallel to the IBS in the xy plane. (g) Formation of an ice layer on top of the vicinal ice layer, and (h) growth of the ice nucleus into a 3D ice nanocrystal. The water molecules in the liquid are represented by red spheres, whereas those in the ice-like layers are represented by blue–white spheres.

Previous experiments have shown that AFPs have the ability to tune ice nucleation.^23,42^ We performed four independent MD simulations, all of which showed spontaneous ice nucleation near the IBS of the model TmAFP within several microseconds (see Movie S1 as an example). In contrast, no ice nucleation events were observed near the nonice-binding surface (NIBS) in these simulations, consistent with prior experimental findings.^23^ According to the trajectories, the ice nucleation and growth exhibited a sequential cross-dimensional process, namely, from 1D to 2D and then 3D ice-like structural formation. In the precritical nucleus formation stage, a 1D single-file water chain was observed in the inverted groove of the IBS, where the CW formed hydrogen bonds with the hydroxyl groups on threonine residues and the carbonyl groups on cysteine residues (Figure 1a). As depicted in Figure 1b, these CW molecules were linearly distributed, mostly located at the coordinates of 2.91, 3.37, and 3.82 nm along the *x*-axis with an intermolecular spacing of approximately 0.45 nm. This spacing arrangement closely aligns with the lattice constant of ice Ih.^43^ The dipole orientation of the CW was centered around 90° throughout the simulation, indicating that highly ordered water molecules were firmly anchored within the groove (see Figure 1c and Movie S2).

In the next stage, a 2D ice layer formed on the IBS (Figure 1d). The IBS presents a flat pattern of T*x*T repeats, with T representing threonine and *x* representing a variable amino acid. As shown in Figure 1e,f, the average distances within the threonine-cysteine-threonine (TCT) motif hydroxyl groups and between adjacent TCT motifs (a_OH_ = 0.74 nm, b_OH_ = 0.46 nm)^44^ closely resemble the spacing between oxygen atoms in the ice lattice (a_ice_ = 0.73 nm, b_ice_ = 0.45 nm). This close resemblance, with a slight deviation of 0.01 nm, suggests that the ordered array of threonine side chains and the constrained CW mimic an ice surface, thereby promoting ice nucleation. Indeed, the CW and the hydroxyl groups on the IBS can act as hydrogen bond acceptors and donors, respectively, as evident from the snapshots in Figure 1d. In the six-molecule ring, three water molecules form hydrogen bonds with the IBS hydroxyl groups or CW (depicted in blue–white), whereas the remaining three molecules are hydrogen bonded with neighboring water molecules (depicted in yellow–white). The formation of two-dimensional quasi-ice layers was also detected in the TIP4P/ICE and TIP5P water model systems (Figure S1). Similar 1D CW plus a 2D ice layer has been reported as an anchored clathrate or ice-like motif in previous studies for binding with ice and the protein.^28,45,46^

**Figure 2:**
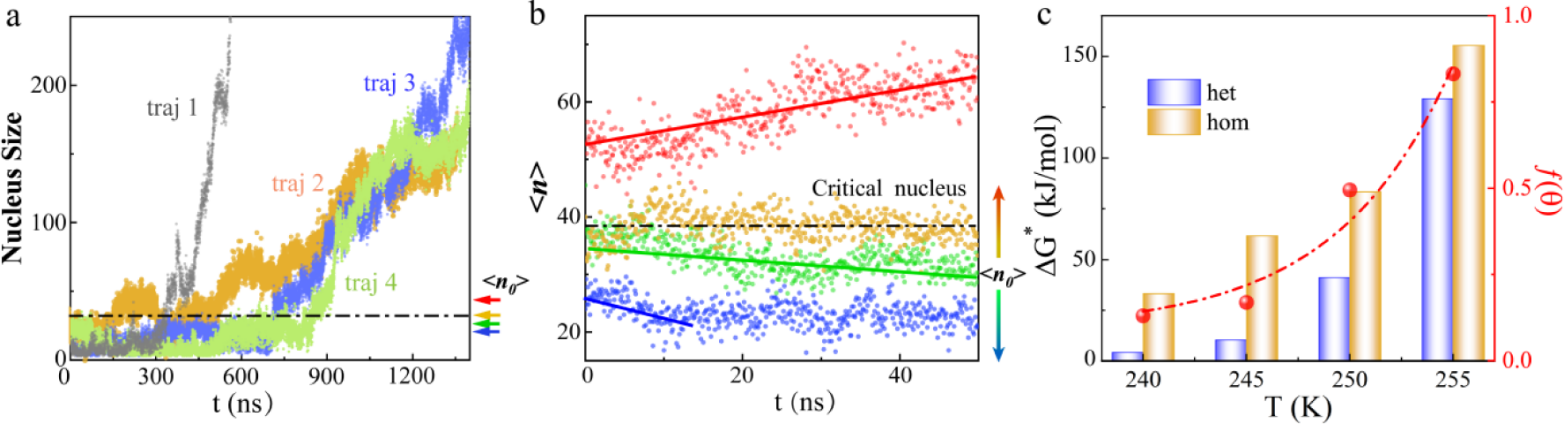
Critical nucleus size and free energy barrier near the IBS of the protein. (a) Nucleus size (number of water molecules in the largest ice nucleus) versus the simulation time for four independent MD simulation trajectories. (b) Nucleus size averaged over 20 independent simulations versus the simulation time. Note that the initial nucleus size < *n*_0_> values, as marked by the four different colored arrows in (a), were obtained from the four original MD trajectories. (c) CNT prediction of the influence of TmAFP on lowering the nucleation barrier and the potency factor *f* of heterogeneous nucleation relative to homogeneous nucleation versus temperature (the dashed line represents the nonlinear polynomial curve fitting).

The microscopic mechanism of the transition from a 2D ice nanolayer to a 3D ice nucleus was explored. The region above the IBS can be divided into three sections, and each section can accommodate only a single layer of water molecules, as shown in Figure S2a. The first ice-like water layer emerged at ∼100 ns, as shown in Figure S2b, indicating that the IBS of TmAFP can rapidly induce a locally ordered 2D water layer. The second ice-like layer subsequently emerged at ∼400 ns, and thereafter, the third layer became more structured at ∼500 ns. During the MD simulation, we tracked the q_6_ parameter,^47^ which is a commonly used and highly sensitive index for monitoring the orientational order. We focused on whether the relative orientation of individual water molecules was coherently ordered. Here, q_6_ for the first water layer increased, as shown in Figure S2c, and that for the second layer exhibited a positive trend after 200 ns. However, the q_6_ for the third layer displayed no significant change. These results showed that AFPs induced ordering of water molecules on the IBS template in a layer-by-layer fashion. Moreover, the hydrogen bond lifetime of the first 2D ice layer and the overlying water molecules was monitored. The result was represented by the intermittent time correlation function C_H_(t) of the hydrogen bond, which was computed every 50 ns during the MD simulations. In general, C_H_(t) exhibited an attenuation trend. However, the attenuation trend became weaker in later stages of the simulation, suggesting a continuous increase in the hydrogen bond lifetime, as illustrated in Figure S3a. Additionally, Figure S3b illustrates the probability (Γ_HB_) at time t that a pair of initially bonded water molecules remained bonded, which was calculated by integrating and normalizing C_H_(t) with respect to the correlation time. Γ_HB_ converged to 0.9 at 550 ns, indicating that more than 90% of the hydrogen bonds remained intact within a 20 ns correlation time. This result indicated that “long-lasting” hydrogen bonds formed along with the first and second ice layers, followed by expansion of the ice core, as illustrated by Figure 1 g,h and traj2 in Figure 2a.

We examined the size evolution of the ice nucleus on the IBS in four independent simulations, as illustrated in Figure 2a. Here, the largest ice nucleus exhibited both cubic and hexagonal ice-like structures, excluding those water molecules at the interface with liquid water. In each MD trajectory, the nucleus size (*n*) showed continuous fluctuations for *n* < 40, as indicated by the dotted line. However, once *n* exceeded 50, the ice nucleus began to steadily grow. We selected four distinct nucleus sizes around *n* ∼ 40 (*n*_0_: ∼ 28, ∼ 38, ∼ 40, and ∼50), as marked by arrows of different colors in Figure 2a, and used the seeding approach to identify the critical nucleus size.^48,49^ To this end, we performed 20 independent simulations for each nucleus size, as shown in Figure 2b. Nuclei originating from seeds exceeding the critical size tended to grow (d⟨*n*⟩/dt >0), whereas those originating from seeds less than the critical size tended to shrink (d⟨*n*⟩/dt <0). These observations suggest that the critical size of the nucleus, consisting of ice Ih/Ic-like water molecules, is approximately 38. Notably, this critical size is significantly smaller than that for homogeneous nucleation of water at the same temperature, which involves ∼80 ice Ih/Ic-like water molecules, as shown in Figure S4. Our findings indicate that the IBS can indeed act as an effective ice nucleation surface to reduce the critical size, thereby facilitating ice formation.

The working principle for the AFPs inducing ice nucleation can be attributed to the lowered free energy barrier due to the IBS of the protein. We utilized CNT to estimate the free energy barrier for nucleation and growth of ice nuclei on the IBS, as described in the Methods section. At 240 K, the free energy barrier was approximately 4.3 kJ/mol, which was estimated with a typical line tension value τ = 5 pN.^28^ Note that this barrier is markedly lower than that of 33.2 kJ/mol for homogeneous nucleation, as shown in Figure 2c. More specifically, the critical size of the ice embryo and the associated free energy barrier were analyzed and computed to further clarify the ice nucleation enhancement efficiency at different temperatures, as shown in Figures S4 and 2c. We characterized the ability of the IBS to lower the critical free energy barrier by *f* = Δ*G*_Het_^*^/Δ*G*_Hom_^*^, where Δ*G*_Het_^*^ and Δ*G*_Hom_^*^ refer to free-energy barriers of heterogeneous and homogeneous nucleation, respectively. As the temperature increases, the effectiveness of TmAFP in lowering the critical free energy barrier diminishes (Figure 2c). The freezing process of water, as induced by TmAFP in cryogenic settings, can be divided into several stages: (i) 1D CW molecules are immobilized and organized within the inverted trough. (ii) The hydroxyls of TCT motifs and the restricted CW molecules act together as an ice-like surface, inducing the formation of a 2D ordered ice layer on the IBS. (iii) The 2D ice layer can further result in reorganization of water molecules into ice. In other words, the surface of the IBS can significantly lower the free energy barrier of nucleation, facilitating the formation of a 3D ice crystal.

### Role of Functional Groups at the Ice-Binding Site in Promoting Icing

**Figure 3:**
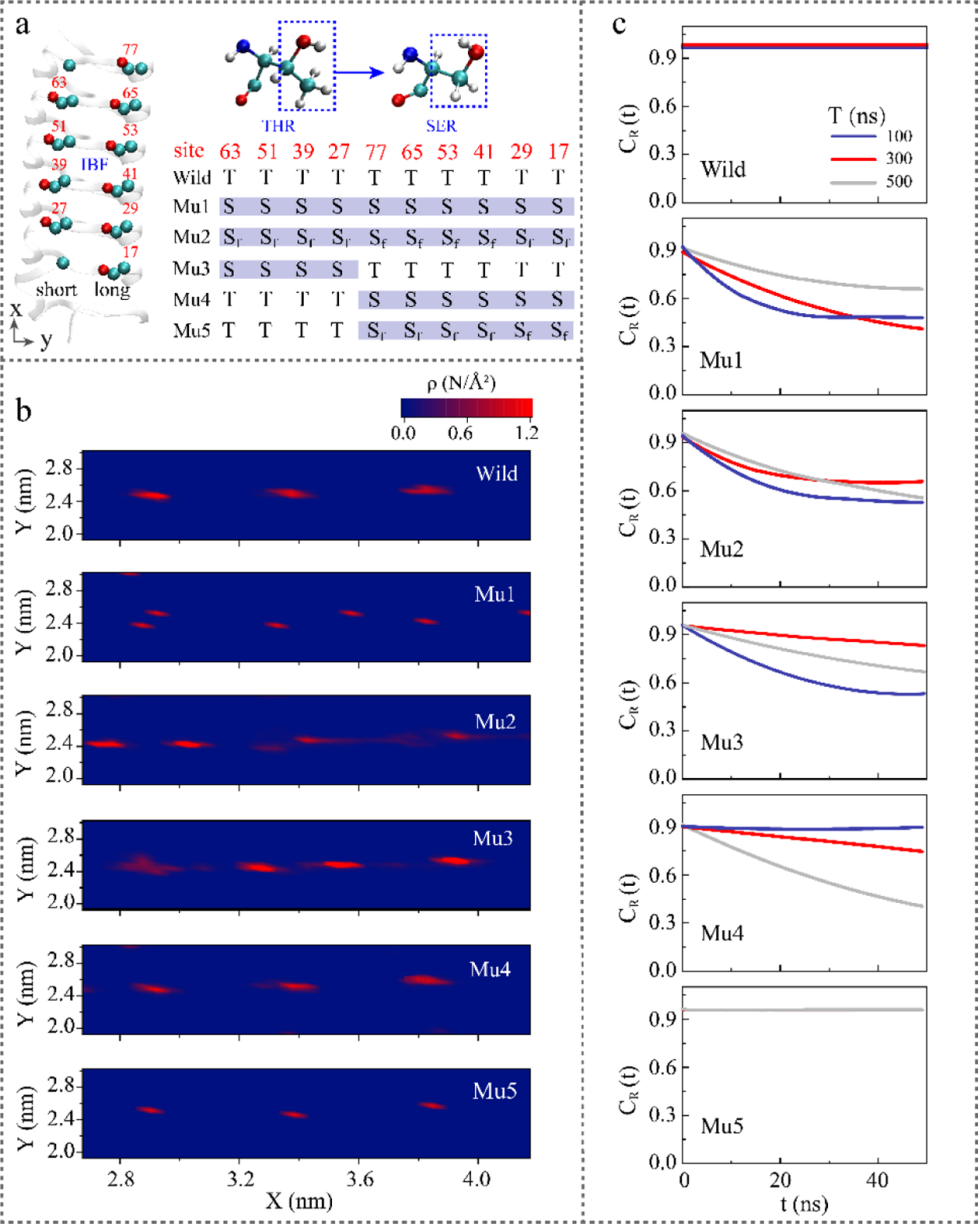
Effects of mutations at the ice-binding site of TmAFP on its ice-binding ability. (a) Ice-binding site of TmAFP. The ice-binding site contains a row of four threonine residues (T27, T39, T51, and T63), which we call the short side, and a row of six threonine residues (T17, T29, T41, T53, T65, and T77), which we call the long side. The hydroxyl and methyl groups of threonine at the ice-binding site are shown with red and cyan balls, respectively. Mutation of threonine to serine via removal of methyl. The table shows the residues at the IBS in wild-type AFPs and five different kinds of mutant-type proteins. The threonine and serine residues are expressed as T and S, respectively. S_f_ stands for the fixed serine, and the mutation sites are highlighted in blue shading. (b) Density distribution of CW molecules during the simulation time of 0.1–500 ns, and (c) residence time correlation function (C_R_(t)) of CW molecules within 50 ns at 100, 300, and 500 ns for wild-type and mutant-type proteins.

Note that the IBS possesses both hydrophilic hydroxyl groups and hydrophobic methyl groups. A main goal of our research is to understand how the synergy between hydrogen bonding and hydrophobic interactions^50,51^ on the IBS influences the nucleation of ice embryos. Figure 1a,d–f illustrates that the spacing between hydrophilic hydroxyl groups is similar to that between water molecules in ice, thereby promoting ice-like ordering on the IBS. Previous studies have suggested that hydrophobic interactions are also required for molecular recognition of and adherence to ice; in particular, the methyl groups play an important role in stabilizing the ice-binding site and inducing a locally low-density hydration layer through hydrophobic interactions, as well as by slowing the water dynamics in the trough of the IBS.^18,52^ To gain deeper insight into the impact of each functional group of the IBS on ice nucleation, a series of artificial mutations were performed, as depicted in Figure 3a. Structural visualization diagram of the wild-type and mutant-type proteins was shown in Figure S5.

Initially, all threonine residues were substituted with serine by removing the methyl groups from the ice-binding site (named Mu1). The structural stability of the ice-binding site is crucial for the site to effectively function in its role of interacting with ice. For threonine residues, the hydroxyl groups exhibited consistent orientations, whereas for serine residues, they were deflected, as depicted in Figure S6. This behavior is more vividly illustrated in Movies S2 and S3, which show animations of the IBS with CW molecules of the wild type and Mu1 type. Furthermore, the distribution of the angle Φ_N–C–C-OH_ at the ice-binding site (Figure S7a inset) was nearly uniform for T39, T41, T51 and T53 of the wild-type TmAFP, centered around 59°. In contrast, for the mutant-type serine residues at the same site, the distribution of Φ_N–C–C-OH_ was centered at 88°, 97°, 160° and 173°, respectively, as shown in Figure S7a. The distance between oxygen atoms of equivalent hydroxyls in TCT motifs across adjacent loops, d_O–O_, as depicted in Figure S7b, indicated significant vibrational changes in serine residues around their crystallographic positions after mutation. Specifically, the dihedral angle of S39 was deflected between 310 and 370 ns, leading to an increase in d_O–O_ from 0.74 to 0.97 nm, which deviated from the ice lattice spacing. These findings align with those of prior studies, indicating that methyl groups in threonine residues strengthen the conformational stability of the ice-binding site while enhancing its ability to induce ice-like order in solution.^18^ However, whether the decreased stability of the CW in Movie S3 is due to the instability of hydroxyl groups needs further investigation.

The hydroxyl groups were subsequently fixed after mutating threonine to serine (named Mu2), establishing a 2D lattice-like configuration. This artificial arrangement featured distances between hydroxyl groups close to those between water molecules in ice, aiming to mitigate the influence of unstable hydroxyl groups. Although the lattice alignment of hydroxyl groups could facilitate hydrogen bonding with water, the aligned hydroxyl groups alone were insufficient to induce ice nucleation due to the absence of stable and ordered CW molecules (Figure 3b,c). The result suggested that methyl plays an important role in inducing freezing, and its mechanism needs further exploration. Note that the ice-binding sites comprised a row of six threonine residues (T17, T29, T41, T53, T65, and T77), referred to as the long side, and a row of four threonine residues (T27, T39, T51, and T63), termed the short side, as shown in Figure 3a. The methyl groups on the short side and the hydroxyl groups on the long side were positioned along the inner boundary of the ice-binding sites, facing the trough. The influence of functional groups at different locations was further investigated by mutating the residues on the short side (named Mu3) and the long side (named Mu4) to serine.

**Figure 4:**
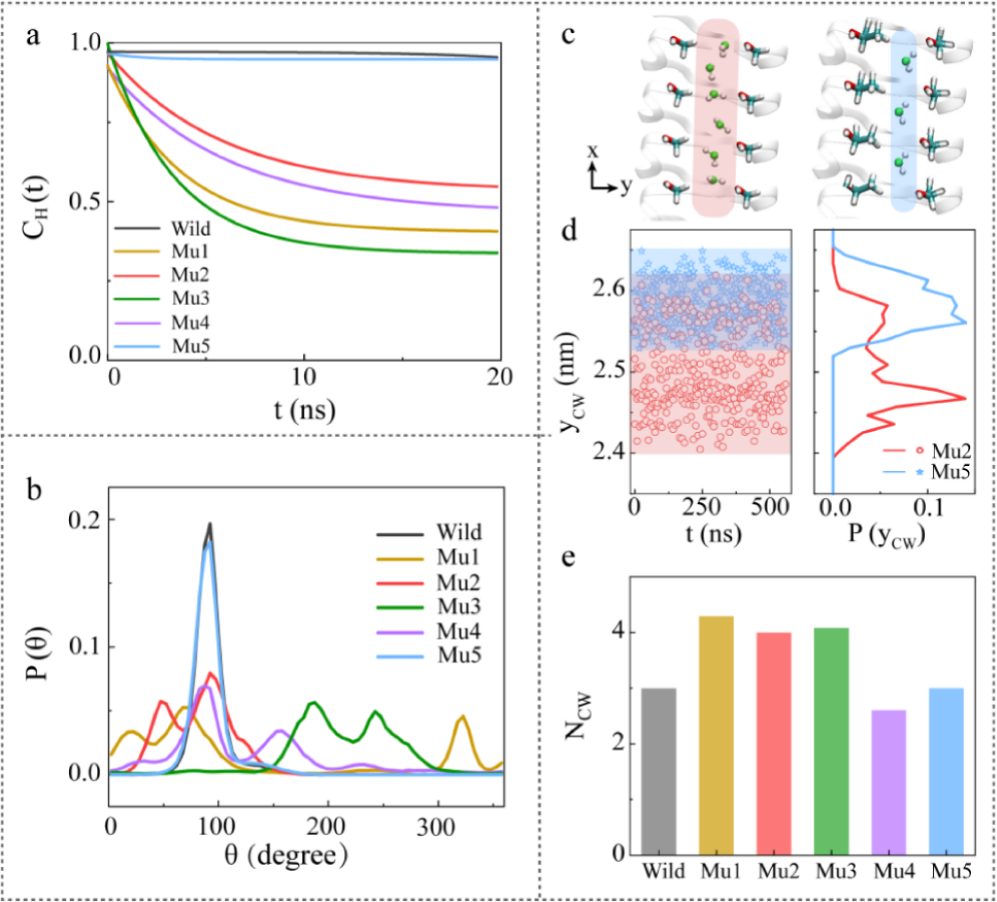
Analysis of the effects of functional groups on the IBS. (a) Hydrogen-bond lifetime correlation functions C_H_(t) between CW molecules and the hydrogen groups on the IBS. (b) Dipole angle distribution of CW molecules in the inverted trough. The data are based on the trajectory over a period of 500 ns, where, for the C_H_(t) and P(θ) of CW molecules, only the last 20 ns of data were counted. (c) Snapshots of the CW molecules in the inverted trough of Mu2 type and Mu5 type. (d) Projected distance along the *y*-axis between CW molecules and the hydroxyl groups on the long side (y_CW_), as well as probability distributions of these distances. The shaded areas in (c) and (d) highlight the regions where CW molecules are predominantly located. (e) Average occupancy number of CW molecules in inverted trough.

There was no discernible pattern for mutant-type Mu3, as evident from the density distribution in Figure 3b and snapshots of the IBS with CW molecules at different simulation times in Figure S6. However, the CW molecules were situated at specific locations within the inverted trough, with a centroid distance of approximately 0.45 nm observed for mutant-type Mu4 (Figure 3b). The C_R_(t) for CW molecules situated in the inverted trough for Mu4 decreased with increasing residence time, indicating increasing freedom of CW molecules (Figure 3c). We hypothesize that the flipping of the hydroxyl groups on the long side weakened the stability of the CW molecules. Finally, Mu5 was constructed by modifying the residues on the longer side and stabilizing the hydroxyl groups after the mutation. Interestingly, CW molecules in the trough of mutant-type Mu5 exhibited similar behavior to those of wild-type TmAFP. The C_H_(t) in Figure 4a shows that the mutant-type Mu5 proteins could form long-lived hydrogen bonds with CW molecules, similar to the wild-type protein. Moreover, Γ_HB_ was consistently above 0.8 for both the wild type and Mu5 type but significantly greater for the other mutant types, and there was no observable increasing trend over time, as shown in Figure S8. This finding indicates that the role of the methyl groups varies depending on their position, and these groups, particularly the methyl groups on the short side, not only aid in anchoring the ice-binding sites but also are crucial for stabilizing the CW molecules.

In the inverted trough of mutant-type proteins when missing methyl groups on the short side, which increased freedom of CW molecules as shown in Figures 4a,b and S9. This occurs because the CW molecules had a broader space for mobilization, as depicted in Figure 4c,d, along with the increased capacity of the trough to accommodate more water molecules, as shown in Figure 4e. Consequently, there were more CW molecules within the troughs of mutant-type Mu1, Mu2, and Mu3 than within those of the wild type, Mu4 and Mu5. We also noticed that Figure 3b demonstrates that CW molecules within the troughs of mutant-type Mu4 remained anchored at specific sites, regardless of the flipping of the hydroxyl groups on the long side. This result suggests that the position of CW molecules is predominantly controlled by the carbonyl groups associated with cysteines.

Finally, biomimetic graphene surfaces were engineered by attaching amino acid residues. As depicted in Figure S10, two different model surfaces were developed: one with threonine grafted and another with both threonine and cysteine grafted. The model surface modified solely with threonine formed amorphous ice at low temperature, whereas the model surface incorporating both threonine and cysteine facilitated the formation of hexagonal ice. This occurs because the presence of cysteine creates a retention site for CW, as illustrated in Figure S10, which is orderly arranged, thereby facilitating the formation of hexagonal ice on the surface. These results not only confirm that ice formation is catalyzed by the IBS of TmAFP but also highlight the crucial role of cysteines in providing anchorage for enhancing the stability of CW molecules on these biomimetic surfaces. Overall, the methyl groups not only enhance the conformational stability of the ice-binding site but also help confine the CW molecules within the trough through spatial restriction. Additionally, the hydroxyl groups not only provide structural matching to the lattice of ice but also facilitate hydrogen bond formation and stabilize the CW molecules.

## Conclusion

Ice nucleation on TmAFP was successfully simulated via all-atomistic MD simulations. These simulations revealed a prenucleation process with the formation of 1D CW and then 2D nanoice, followed by heterogenous nucleation on the 2D nanoice toward a 3D ice nucleus. More specifically, the atomistic-level mechanism for ice nucleation on the IBS can be summarized as follows. Initially, methyl groups on the short side confine the water in the channel. The confined water is subsequently immobilized due to the formation of hydrogen bonds with both the carbonyl groups in the backbone and the hydroxyl groups on the long side of the ice-binding site. These CW molecules, in conjunction with the hydroxyls, form an array on the IBS that can induce the formation of the first 2D ice layer, which then triggers ice nucleation. Furthermore, we demonstrate that the IBS can reduce the critical nucleus size and lower the free energy barrier for ice nucleation, thereby facilitating ice formation. To our knowledge, this is the first simulation of ice nucleation on nanoscale model surfaces with a fully atomistic model, which will help achieve an atomistic level understanding of the promotion effect of TmAFP on ice nucleation and guide future research on biosystem-related heterogeneous ice nucleation.

## Methods

### MD Simulations

The MD simulations were performed using the GPU-accelerated GROMACS-5.0.7 software suite. The simulation supercell had a size of 6.325 nm × 4.694 nm × 30 nm and contained a TmAFP (PDB ID code: 1EZG) solvated in water with 6887 water molecules. Two counterions were added to neutralize the system. The IBS of TmAFP consists of two rows of threonine residues, as shown in Figures 1a and 3a. Periodic boundary conditions were implemented in the plane of the ice surface. The simulation was performed in the canonical (NVT) ensemble with a velocity-rescale thermostat at 240 K. The simulation step was 1.0 fs. The all-atom CHARMM27 force fields of proteins were used in the MD simulations.^53^ The water model was a six-site model (TIP6P), which has been shown to allow relatively fast spontaneous ice nucleation, typically within a few hundred nanoseconds.^54^ The backbone of the proteins was fixed during the MD simulations. An energy minimization process of 1000 steps using the steepest descent algorithm was undertaken first, followed by MD simulations until ice nucleation and growth occurred on the IBS. In addition, another five mutant-type proteins, as detailed in Figure 3a, were used to investigate the role of methyl groups on the IBS in ice nucleation.

### Prediction of the Nucleation Free Energy Barrier via Classical Nucleation Theory

The free energy barrier related to homogeneous nucleation^55^ is given by
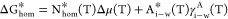

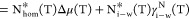
where  is the size of the critical nucleus. Δμ(T) represents the excess chemical potential of the water with respect to the ice,  and  represent the area and molecular number of the ice–water surface, respectively, and γ_i−w_ represents the surface tension of the ice–water surface. The superscripts A and N represent the unit area and number of water molecules, respectively.

The free energy barrier related to the heterogeneous nucleation of ice on a surface^24^ is given by
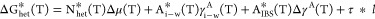

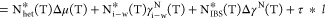
where  is the size of the critical nucleus on the IBS and  and  are the area of and number of molecules on the IBS, respectively. Δγ represents the difference between the surface energies of the crystal surface and liquid surface, and the superscripts *A* and N represent the unit area and number of water molecules, respectively. τ is the line tension of the surface–crystal–liquid interface. We neglected the temperature dependence of τ while using a typical value of τ = 5 pN.^28^ Here *l* is the length of the contact line of the three-phase crystal–liquid–surface interface. The relevant thermodynamic properties of the interfacial system are shown in Table I (for more details, see the Supporting Information).

**Table I:** Relevant Thermodynamic Properties of the Interfacial System (Units: kJ/mol)

Temp	240 K	245 K	250 K	255 K
μ_ice_	–50.19	–50.20	–50.21	–50.23
μ_Wat_	–49.32	–49.48	–49.66	–49.85
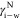	0.97	1.11	1.25	1.37
Δγ^N^	–3.48	–3.16	–2.86	–2.56

## Supporting Information Available

The computational details encompass the identification of the ice nucleus and the characterization of parameters related to water molecular dynamics, including hydrogen-bond lifetime, orientation distribution of water dipoles, and water residence time; key parameters for predicting the nucleation free energy barrier include the identification of interfacial ice molecules, the chemical potential difference between hexagonal ice and liquid water, the surface energy of the ice–water interface, and the difference in surface energies between the crystal and liquid surfaces (PDF)
Simulated ice nucleation process on the IBS of TmAFP with the protein’s main chain immobilized (MOV)
Simulated dynamic behavior of confined water within the threonine groove on the IBS of native TmAFP protein (MOV)
Simulated dynamic behavior of confined water within the serine groove on the IBS of Mu1 protein (MOV)

## Supplementary Material

ja4c15210_si_001.pdf

ja4c15210_si_002.mov

ja4c15210_si_003.mov

ja4c15210_si_004.mov
