# Supplementary material for: Fully Atomistic Molecular Dynamics Simulation of Ice Nucleation Near an Antifreeze Protein
Source: J Am Chem Soc. 2025 Jan 23;147(5):4411–8. doi: 10.1021/jacs.4c15210 (PMC11803617; doi:10.1021/jacs.4c15210)
Supplement: Supplementary file 1 — ja4c15210_si_001.pdf [file ja4c15210_si_001.pdf]

Supporting information

# Fully Atomistic Molecular Dynamics Simulation of Ice Nucleation near an Antifreeze Protein

Yue Zhang[1#], Ning Wei[1#], Liwen Li[2,3#], Yuan Liu[4], Changxiong Huang[3], Zhen Li[1], Yujie Huang[5], Dengsong Zhang[5*], Joseph S. Francisco[6*], Junhua Zhao[1*], Chunlei Wang[5*], Xiao Cheng Zeng[3*]

[1] Jiangsu Key Laboratory of Advanced Food Manufacturing Equipment and Technology, Jiangnan University, Wuxi 214122, China

[2]School of Petroleum Engineering, China University of Petroleum (East China), Qingdao, 266580, China

[3] Department of Materials Science and Engineering, City University of Hong Kong, Kowloon, Hong Kong, 999077 China

[4] School of Chemical Engineering and Technology, Sun Yat-Sen University, Zhuhai 519082, China

[5] International Joint Laboratory of Catalytic Chemistry, Innovation Institute of Carbon Neutrality, College of Sciences, Shanghai University, Shanghai 200444, China

[6] Department of Earth and Environmental Science, University of Pennsylvania,

Philadelphia, PA 19104, USA

[#]These authors contributed equally: Y. Z., N. W., L. L.

[*]Corresponding author. E-mail:

dszhang@shu.edu.cn (D. Z.)

frjoseph@sas.upenn.edu (J. S. F.)

junhua.zhao@163.com (J. Z.)

wangchunlei1982@shu.edu.cn (C. W.)

xzeng26@cityu.edu.hk (X. C. Z.)

## Identification of ice nucleus

We used the bond orientational order parameter of water molecules to identify the ice nucleus. The bond orientational order parameter $q_l$ for a given water molecule is defined as:

$$q_l = \left[\frac{4\pi}{2l+1}\sum_{m=-l}^{+l}|\bar{q}_{lm}|^2\right]^{1/2} \quad (1)$$

where $\bar{q}_{lm}(i) = \frac{1}{N_B}\sum_{i=1}^{N_B}\gamma_{lm}(\theta_i(r),\phi_i(r))$ , and $\gamma_{lm}(\theta_i(r),\phi_i(r))$ are spherical harmonics of rank $l$ and $m$, while $N_B$, $\theta_i(r)$ and $\phi_i(r)$ are the bond number of oxygen atoms closest to $i$ atom within 0.35 nm ($N_B \le 4$) and the two polar angles of each of the $N_B$ bonds between a central water ($i$) and the four closest water or hydroxyl groups[1, 2].

The CHILL+ method was used to identify water molecules belonging to either the liquid or ice phase, based on the correlation of orientational order of a water molecule with its $N_B$ closest neighbors as discussed above [3, 4]. A $2l + 1$ dimensional vector $\vec{q_l} = [\bar{q}_{l,\ -l}, \bar{q}_{l,\ -l+1}, \ \ldots, \bar{q}_{l,\ l-1}, \bar{q}_{l,\ l}]$ was constructed for each atom $i$. We then computed the correlation function, $\mathrm{C(i,j)}$:

$$\mathrm{C(i,j)} = \frac{q_l(i)\cdot q_l(j)}{|q_l(i)||q_l(j)|} = \frac{\sum_{m=-l}^{l} q_{lm}(i)q_{lm}^*(j)}{\left(\sum_{m=-l}^{l} q_{lm}(i)q_{lm}^*(i)\right)^{1/2}\left(\sum_{m=-l}^{l} q_{lm}(j)q_{lm}^*(j)\right)^{1/2}} \quad (2)$$

where $q_{lm}^*$ is the complex conjugate of $q_{lm}$. CHILL+ identifies liquid when $\mathrm{C(i,j)} \le 0.57$, and ice when $\mathrm{C(i,j)} \ge 0.57$ with $l = 6$, as seen in Figure S11, a criterion consistent with literature references [5, 6, 7]. Here, the cutoff value of 0.57 was shown to provide a reasonable distinction between ice and supercooled liquid water.

## Hydrogen-bond lifetime

We adopted a standard criterion for determining hydrogen bonds (i.e., a donor-acceptor distance between hydrogen and oxygen is < 3.5 Å and the oxygen−oxygen−hydrogen angle is < 30°[8]). The dynamics of hydrogen bonds is characterized by using the intermittent hydrogen bond time correlation function ($\mathrm{C_H(t)}$), defined as:

$$C_H(t) = \frac{\langle h(0)h(t)\rangle}{\langle h(0)h(0)\rangle} \quad (3)$$

where $h(t)$ is a hydrogen-bond population variable that can be either 1 or 0. If a pair of sites are hydrogen bonded at a particular time t, then $h(t) = 1$, otherwise it is 0. The angular brackets above denote the average over all the hydrogen bonds formed at different reference initial time [9]. Integrating and normalizing $C_H(t)$ over the time gives the probability for stability of hydrogen bond with the simulation time:

$$\Gamma_{HB} = \frac{1}{t}\int_0^t C_H(t)dt \quad (4)$$

**Orientation distribution of water dipoles**

Water rotational motion was measured via the orientation of water dipole, defined as the angle θ between the vector joining the oxygen atom of a tagged water molecule to the center of the line joining its two hydrogen atoms on the IBS (xy plane) and the axis of the protein (*x*-direction)[10]. The probability distribution of the angle θ is given by

$$P(\theta) = \sqrt{\frac{1}{N}\sum_{i=1}^{N}(P_i - \langle P_i\rangle^2)} \quad (5)$$

where N = 360 is the total number of angular intervals used in this study, $P_i$ represents the probability of the dipole orientation (angle θ) falling into the $i^{th}$ interval, and $\langle P_i\rangle$ is the average value of the probability[11].

**Identification of interfacial ice molecules**

To determine the position of the $i^{th}$ molecule on the interface of IBS, the ice-water surface, or within the nucleus, the spatial vectors from the $i^{th}$ molecule to its neighboring molecules (within a defined cutoff) were calculated. If the sum of these spatial vectors for the $i^{th}$ molecule approached zero, it was classified as an internal molecule. Conversely, if the sum vector exceeded a certain threshold, the molecule was labeled as a surface molecule (as shown in Figure S12). Then the number of water molecules at the ice-water surface can be obtained by screening the water molecules on the IBS. The specific formula for the sum spatial vector of the $i^{th}$ molecule is given below [12]:

$$r_i^* = (\sum_{j=1}^{n} f(r_{ij}) r_{ijx}, \sum_{j=1}^{n} f(r_{ij}) r_{ijy}, \sum_{j=1}^{n} f(r_{ij}) r_{ijz},) \quad (6)$$

where n and $r_{ij}$ are the number of oxygen atoms in the nucleus, and the spatial vector pointing from i atom to j atom, respectively. $r_{ijx}$, $r_{ijy}$, and $r_{ijz}$ are the components of the vector $r_{ij}$ in x, y, and z directions, respectively. The $f(r_{ij}) = \frac{1-(\frac{r_{ij}}{r_c})^4}{1-(\frac{r_{ij}}{r_c})^{22}}$ was used as a smooth cutoff, and $r_c$ is the cutoff distance.

**Chemical potential difference between hexagonal ice and liquid (Δμ(T))**

The $\mu - T$ curve was obtained based on the Gibbs-Helmholtz relation [13]:

$$(\frac{\mu}{T} - \frac{\mu_0}{T_0})_P = -\int_{T_0}^{T} \frac{H}{T'^2} dT' \quad (7)$$

Here, H is the enthalpy, a function of T, and it was determined by fitting the H obtained from the simulations to a quadratic polynomial for ice, and a third-order polynomial for water as shown in Figure S13a. The chemical potential of ice at 275 K and water at 350 K has already been estimated to be -50.31 kJ/mol and -55.84 kJ/mol [13], respectively. Based on Equation 7, the chemical potentials of ice and water were depicted in Figure. S13b. And the coefficients in the polynomials of H, and the values of $\mu_0$ and $T_0$ were given in Table SI.

**TABLE SI.** Coefficients of the enthalpy H as a function of T, $H = h_0 + h_1 T + h_2 T^2 + h_3 T^3$, with the values of $\mu_0$ and $T_0$ for ice and water. The free energies at other temperatures can be calculated by $\mu(T) = T[\mu(T_0)/T_0 + h_0(1/T - 1/T_0) - h_1 \ln(T/T_0) - h_2(T - T_0)]$. Then the excess chemical potential of the liquid with respect to the crystal $\Delta\mu(T) = \mu_{ice}(T) - \mu_{wat}(T)$.

| | T range | $h_0$ | $h_1$ | $h_2$ | $h_3$ | $T_0$ | $\mu(T_0)$ |
|---|---|---|---|---|---|---|---|
| | K | kJ mol$^{-1}$ | kJ mol$^{-1}$ K$^{-1}$ | kJ mol$^{-1}$ K$^{-1}$ | kJ mol$^{-1}$ K$^{-1}$ | K | kJ mol$^{-1}$ |
| ice Ih | 150-300 | -58.6143 | $4.461\times10^{-2}$ | $3.33598\times10^{-5}$ | | 275 | -50.31 |
| wat. | 237.5-350 | -112.5046 | 0.40893 | $-4.79256\times10^{-4}$ | $-1.087\times10^{-19}$ | 350 | -55.84 |

**Surface energy of the ice-water interface ($\gamma_{i-w}$)**

The mold integration (MI) method was used to calculate the ice-water surface free energy [14]. The MI method involves calculating the reversible work required to induce the formation of a crystal slab in a fluid under coexistence conditions (specifically, at the normal melting temperature, $T_m$= 289 K for the TIP6P model [15]) as shown in Figure S14. This work represents a Gibbs free energy difference, ΔG, and is related to the interfacial free energy by $\Delta G = 2A\gamma_{iw}$, where A is the area of the simulation box side in the direction perpendicular to the mold (the factor of 2 is due to the fact that two interfaces are generated).

A mold of 128 potential wells placed at the oxygen lattice positions of three plane (Basal, PI and PII) in the TIP6P water liquid-ice coexistence conditions. In order to fix the position of the wells, we used the “frozen” GROMACS option. The formation of the crystal slab was induced by switching on an attractive interaction between a mold composed of potential energy wells and the fluid particles. A continuous version of the square-well potential was used to describe well–oxygen interaction [14]:

$$\upsilon_{wo} = -\frac{1}{2}\varepsilon\left[1 - \tanh\left(\frac{r-r_w}{\alpha}\right)\right] \quad (8)$$

where r is the distance between the well center and the oxygen atom with which the well interacts, and α is a parameter that controls the steepness of the potential well, with a value for α = 0.005σ. If the interaction between the wells and the oxygen atoms of the water model is square-well-like (with maximum well depth $\varepsilon_m$ and well radius $r_w$), $\gamma_{i-w}$ can be obtained as:

$$\gamma_{i-w}(r_w) = \frac{1}{2A}\left(\varepsilon_m N_w - \int_0^{\varepsilon_m} d\varepsilon \, \langle N_{fw}(\varepsilon)\rangle_{Np_xT}\right) \quad (9)$$

where $N_w$ is the total number of wells and $\langle N_{fw}(\varepsilon)\rangle$ is the average number of filled wells obtained in the $Np_xT$ ensemble for wells of depth $\varepsilon$. Thermodynamic integration was performed along a path in which the depth of the wells is gradually increased to its maximum value, $\varepsilon_m$.

It should be noticed that the integration in Equation 9 must be reversible. To ensure this, the structure induced by the mold must quickly disappear when the interaction between the mold and the fluid is switched off. Therefore, it is essential to prevent the full formation of the ice slab during integration. This is achieved by performing thermodynamic integration for wells with a radius larger than a specific value $r_w^0$, as seen in Figure S15 there is no continuous core growth when $r_w$> 0.08 nm. This kind of check is shown in Figure S16. All the black points in (a) were obtained by using a fluid configuration as a starting point. Next, we used the final configuration of the simulation with the mold fully switched on ($\varepsilon = \varepsilon_m = 8\,k_BT$) to repeat the calculations. If a crystal slab had been irreversibly formed, we would not obtain the same integrand. The test is satisfactory, though, as seen in (b), and we obtained the red diamonds in (a). In practice, $\gamma_{i-w}(r_w)$ was estimated for several values of $r_w > r_w^0$, and then $\gamma_{i-w}(r_w)$ is extrapolated to $r_w^0$, which is the well radius that provides the correct value of $\gamma_{i-w}$. Finally, we take the average of the three surfaces of ice-Ih as the final surface tension value $\gamma_{i-w}^N = 2.07748\,\mathrm{kJ/mol}$ (Figure S17). Then, we approximate it through Turnbull's heuristic relation of different temperature:

$$\gamma_{i-w}^N(T)/\gamma_{i-w}^N(T_m) = \Delta H_m(T)/\Delta H_m(T_m) \quad (10)$$

where $\Delta H_m$ is the excess enthalpy of the liquid with respect to ice and $T_m$ is the equilibrium melting point of ice.

## Difference between the surface energies of crystal-surface and liquid-surface

The binding free energy $\Delta G_{bind}$ was calculated using umbrella sampling (US) simulations. We use a harmonic tether with force constant of $50\,kcal\,mol^{-1}Å^{-2}$ to control the distance between the center of mass of the protein and ice/water interface. The harmonic tether allows fluctuations of the protein center of mass only in the direction perpendicular to the ice/water interface. The core of the ice is fixed and we control the amount of ice in the simulation cells with a harmonic restraint on the global bond-order parameter Q6 in PLUMED engines, where the equilibrium value of Q6 is determined from the initial configuration of the system. The restraint to maintain the amount of ice is applied to only the mobile water molecules and the harmonic force constant 20 kcal/mol.

We obtained the binding free energy per molecule of the crystal to the IBS, $\Delta G^{N}_{bind} = -4.45$ kJ/mol as seen in Figure S18. Then the difference between the surface energies of crystal-surface and liquid-surface $\Delta\gamma^{N}$ can be written as:

$$\Delta\gamma^{N} = \Delta G^{N}_{bind} + \gamma^{N}_{ice-water} \tag{11}$$

The temperature dependence $\Delta G_{bind}$ using the relation derived [16]:

$$\Delta G_{bind}(T_2) = \Delta G_{bind}(T_1) + \int_{T_1}^{T_2}(-\Delta S_{bind})dT=$$

$$\Delta G_{bind}(T_1)+\int_{T_1}^{T_2}(-S_{IBS-i} + S_{i-w}\cos\theta - S_{w-v})dT =$$

$$\Delta G_{bind}(T_1)+\int_{T_1}^{T_2}(S_{i-w} - S_{w-v})dT \tag{12}$$

where we assumed that water fully wets the IBS of the protein and the surface entropy of the ice-IBS interface is negligible, that is $\cos\theta = 1$ and $S_{IBS-i} = 0$. Here $S_{w-v} = -(\frac{\partial\gamma_{w-v}}{\partial T}v)_{p,A_{w-v}}$ is the surface entropy of the water-vapor interface and $S_{i-w} = -(\frac{\partial\gamma_{i-w}}{\partial T})_p$ is the surface entropy of the ice-water interface[17]. The $\gamma_{i-w}$ was obtained

via Equation 10 as seen in Figure S19a. The $\gamma_{w-v}$ was calculated from the difference between the normal and the lateral pressure $\gamma_{w-v}(t) = \frac{1}{n}\int_0^{L_z}\{p_{zz}(z,t) - \frac{p_{xx}(z,t)+P_{yy}(z,t)}{2}\}dz$ as illustrated in Figure S19b.

**Water residence time**

The residence time of CW molecules located in the inverted groove can be determined via computing the residence time correlation function,

$$C_R(t) = \frac{\langle b(0)B(t)\rangle}{\langle b(0)b(0)\rangle} \tag{13}$$

where $b(0)$ can be either 1 or 0, depending on whether a particular water molecule is in the channel at time $t = 0$ or not; and the variable $B(t)$ is 1 when the tagged water molecule remains in the channel from time $t = 0$ to a later time $t$. The angular brackets indicate the average over all such tagged water molecules and over different time origins. By definition, $C_R(t)$ should provide an estimation of residence time of the CW molecules.

## Supporting Figures

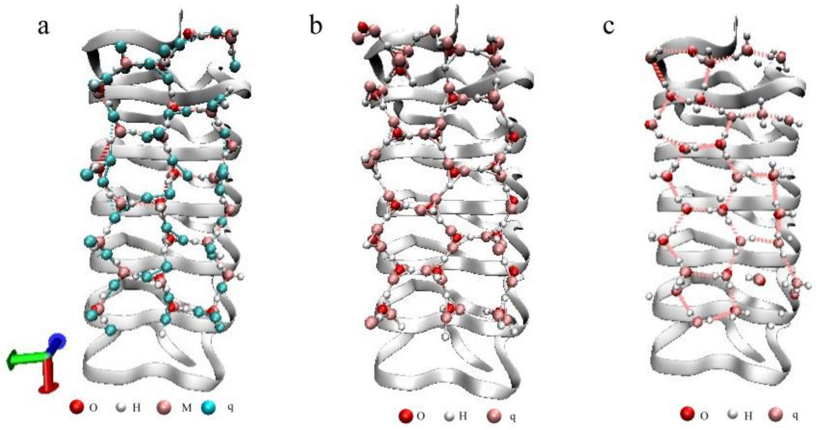


**Figure S1** The ordered ice-like layer on the IBS for **a** TIP6P **b** TIP5P **c** TIP4P/ICE water models.

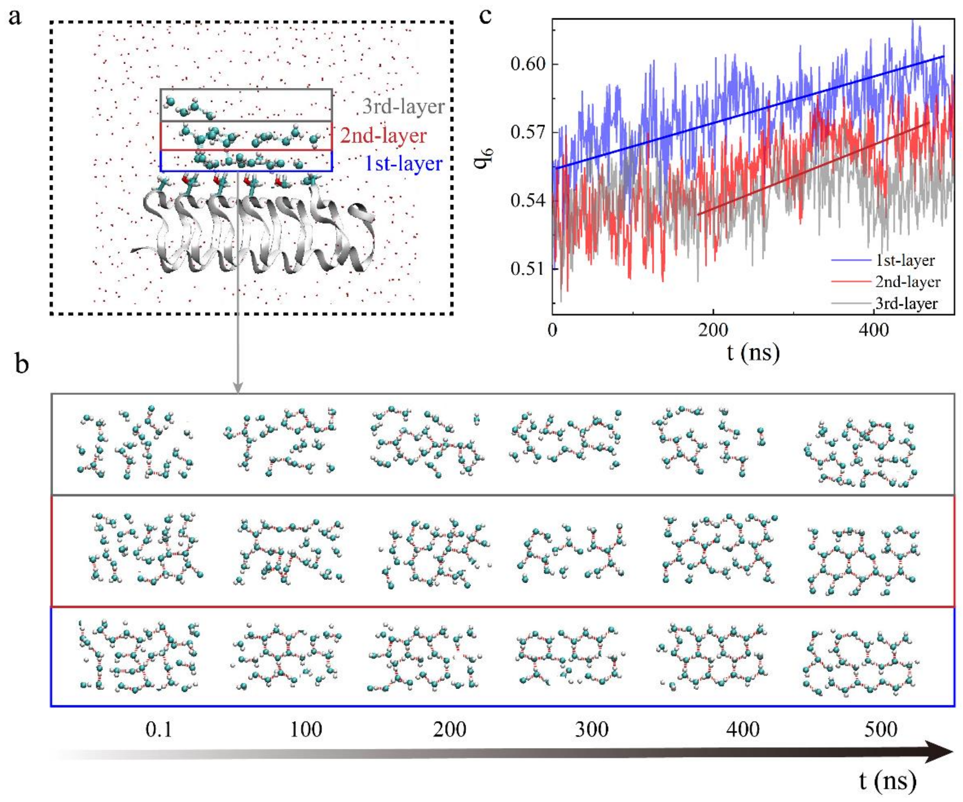


**Figure S2** Time evolution of the water layers. **a** The 1st-layer defined as water molecules within 0.4 nm from the IBS. The same way can be done for defining the 2nd-layer and 3rd-layer. **b** Snapshots illustrating the temporal evolution of the layered interfacial water on the IBS. **c** Time evolution of the order parameter $\mathbf{q_6}$ for the three water layers.

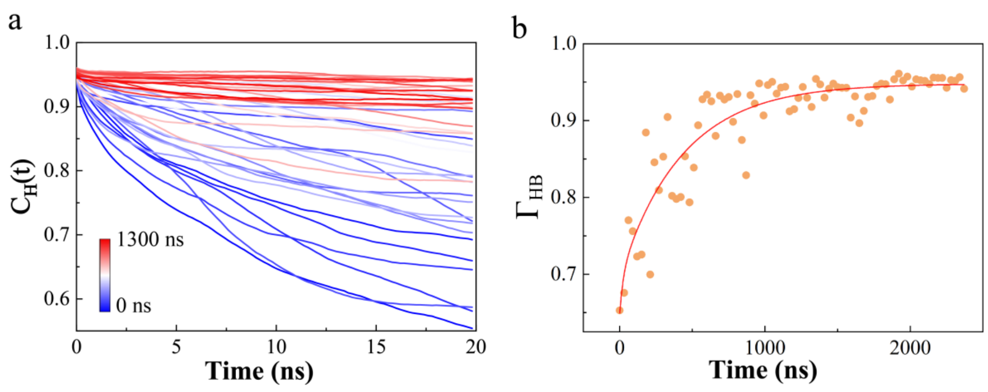


**Figure S3 a** The hydrogen-bond lifetime correlation functions $C_H(t)$. **b** The integrated and normalized hydrogen bond lifetime correlation functions for the quasi-ice bilayer on IBS.

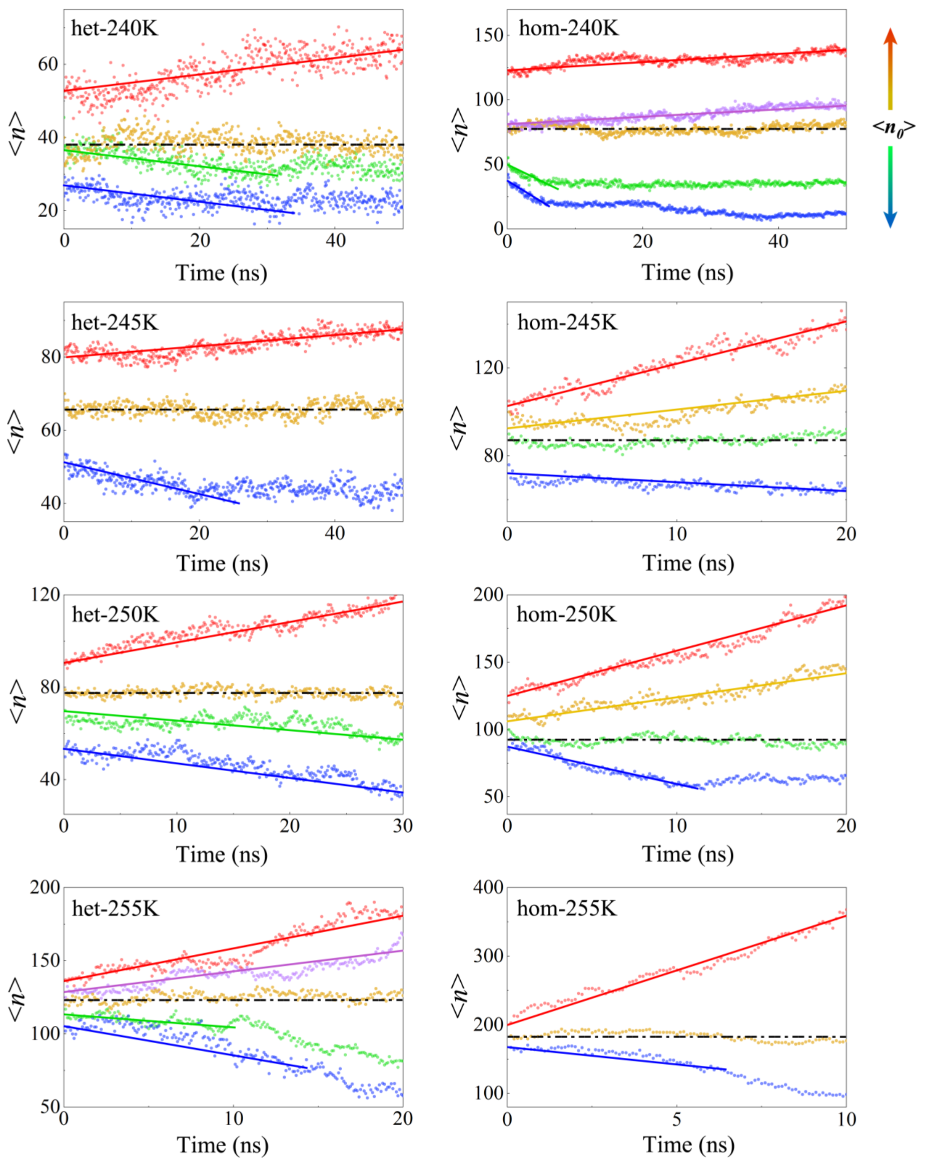

**Figure S4** Number of water molecule in the largest ice nucleus versus the simulation time for homogeneous nucleation and heterogeneous nucleation on the IBS at various temperatures. The critical nucleus for homogeneous nucleation was determined using the seeding method, where artificially pre-formed ice nuclei of various sizes were introduced into a water box. The fluctuations in the sizes of these nuclei were then analyzed to identify the critical nucleus.

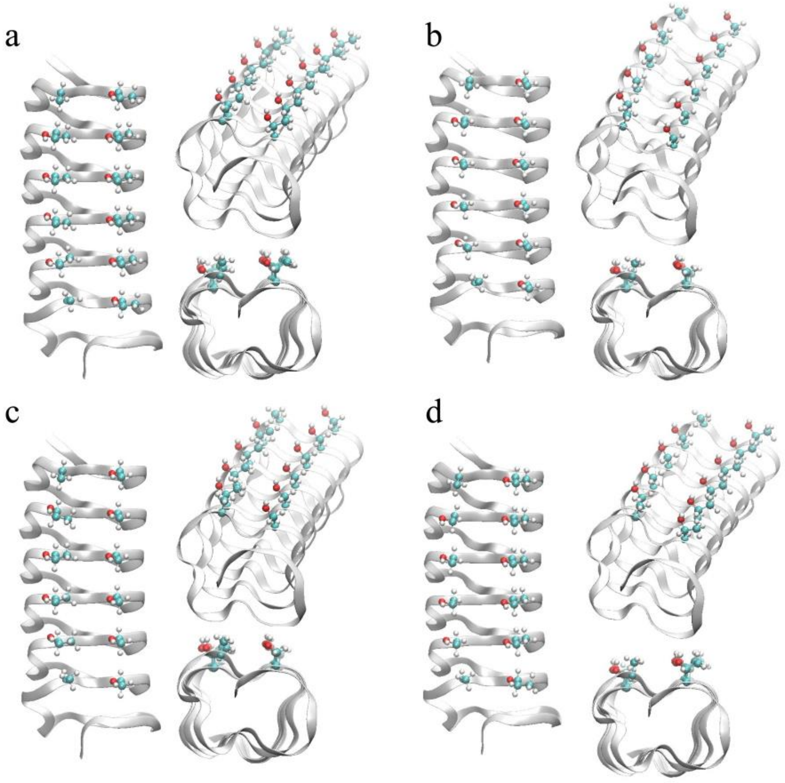


**Figure S5** Structural visualization diagram of the wild-type and mutant-type proteins. **a** Wild; **b** Mu1&Mu2; **c** Mu3; **d** Mu4&Mu5. (It should be noted that the structures of the Mu2 and Mu1 types, as well as the Mu4 and Mu5 types, are identical. However, Mu2 and Mu5 introduce constraints on the hydroxyl group at the mutated amino acid site.)

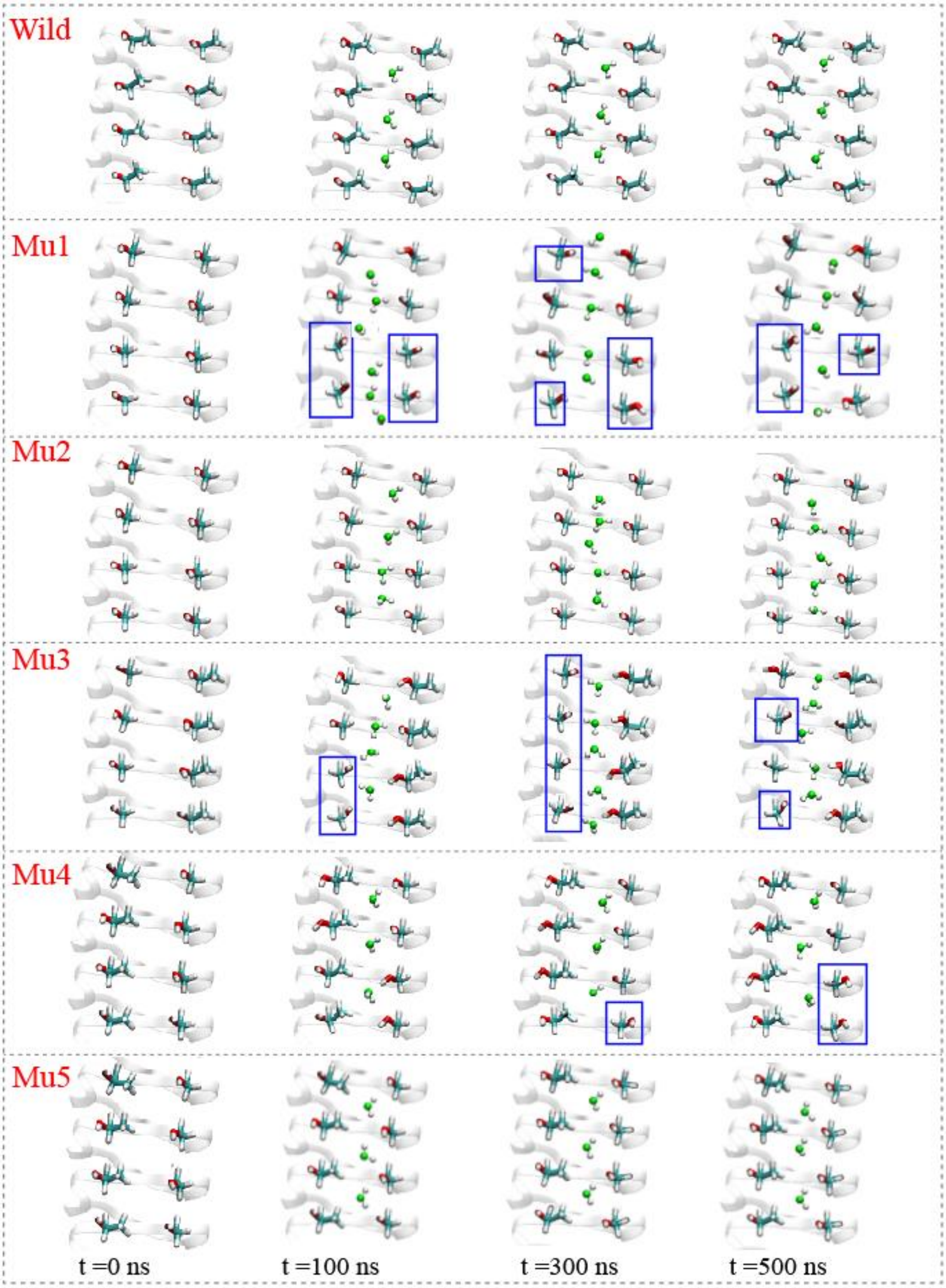


**Figure S6** Snapshots from various simulation times showcase the ice-binding site and CW molecules of both the wild type and mutant type. A blue frame highlights a deflection in the hydroxyl groups at the amino acid site compared to the original snapshot.

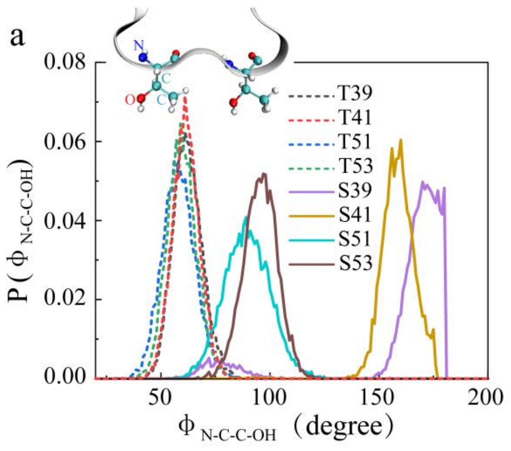


**Figure S7 a** Probability distribution of residues 39, 41, 51 and 53 for the wild-type threonine and mutant-type serine in solution. **b** The distance between oxygen atoms at two adjacent amino acid sites, either T39 and T41 or S39 and S41.

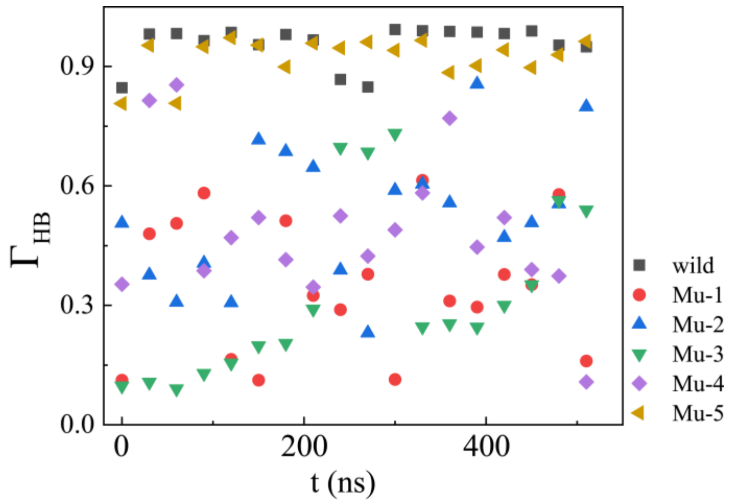


**Figure S8** The normalized average hydrogen-bond lifetime ($\Gamma_{HB}$) reflects the duration of hydrogen bonds between the CW molecules and the hydroxyl groups in Wild-type and Mutant-type proteins. For the Wild-type and Mu5-type, $\Gamma_{HB}$ consistently exceeds 0.8, indicating longer bond durations. However, for other mutant types, $\Gamma_{HB}$ is significantly shorter and does not stabilize as simulation time progresses.

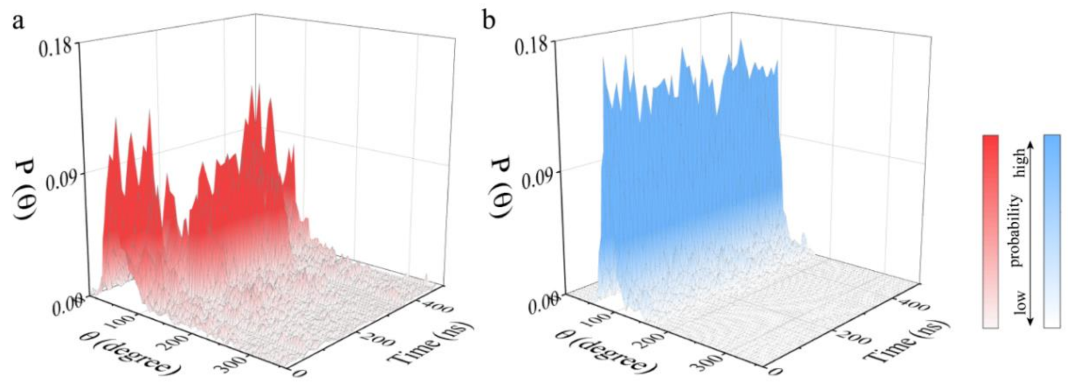


**Figure S9** The dipole angle of CW molecules trapped in the inverted trough versus simulation time of Mu2-type **a**, and Mu5-type **b**.

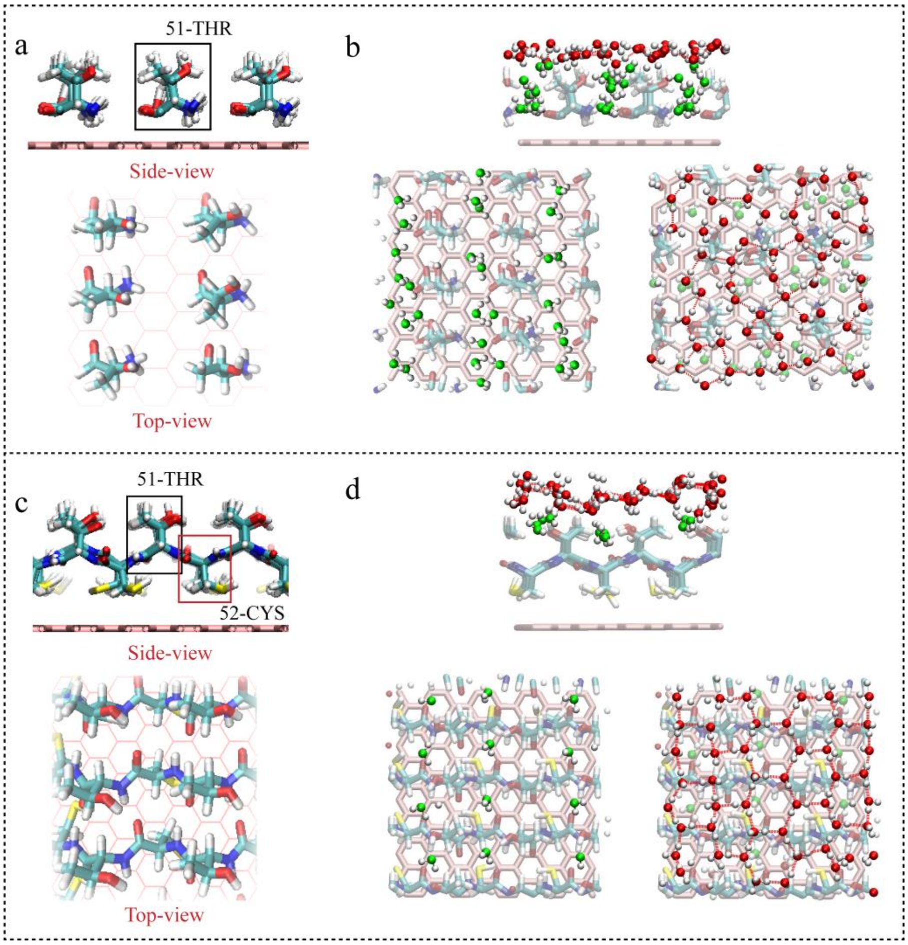


**Figure S10** Preparation of bionic antifreeze protein surface. **a** Graphene surfaces grafted with threonine. **b** Snapshots of channel water and water layer on the biomimetic surfaces after 500 ns. c Graphene surfaces grafted with threonine and cysteine and **d** the structure of water molecule at 500 ns. The amino acids are shown with hollow sticks and the channel water highlighted in green.

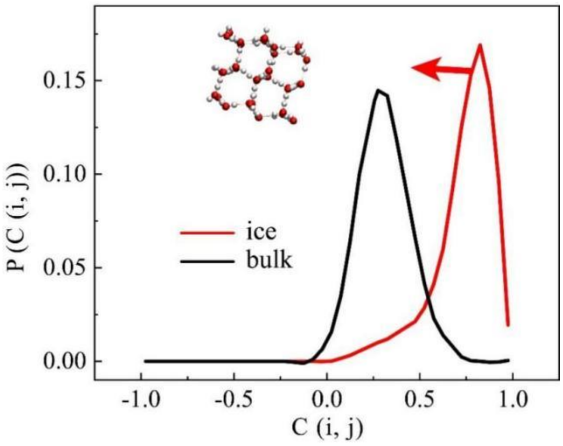


**Figure S11** Ice is identified by the $\mathrm{C(i,j)}$ value of the central water with the four closest water molecules. The distribution of correlation of orientational order parameters $\mathrm{P(C(i,j))}$ from spherical harmonics with $l = 6$, indicating that the $\mathrm{C(i,j)} > 0.57$ for ice.

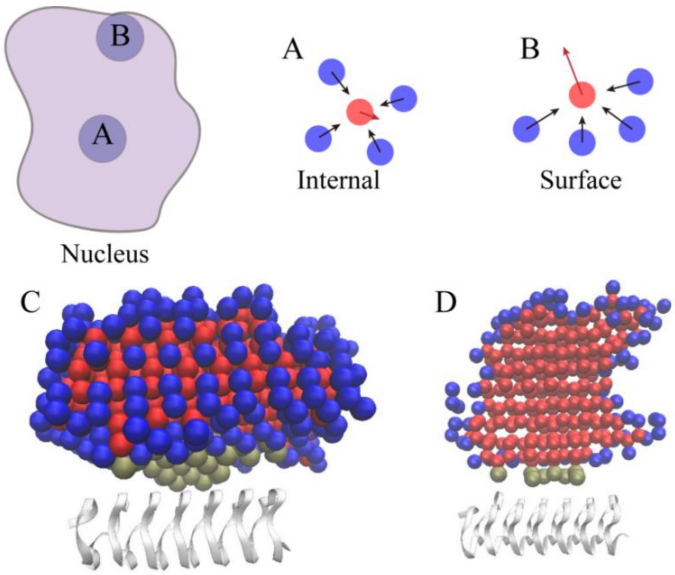


**Figure S12** Schematic diagrams illustrating the differentiation of water molecules in various regions of the nucleus. The vector sum of the local atoms (blue balls) to the central atom (red balls) in the (A) internal and (B) surface nucleus. (C) and (D) are the interfacial (yellow balls), surface (red balls) and internal atoms (blue balls) identified by this algorithm.

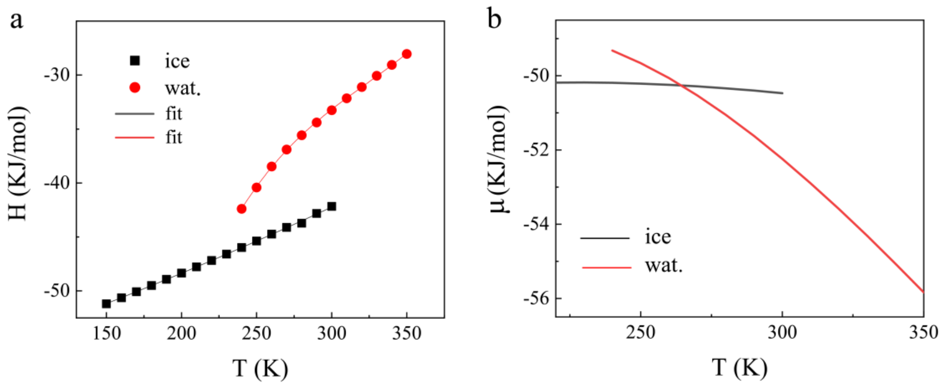


**Figure S13** Computed H-T curves **a**, and the **μ**-T curves **b** based on the TIP6P model for ice and water.

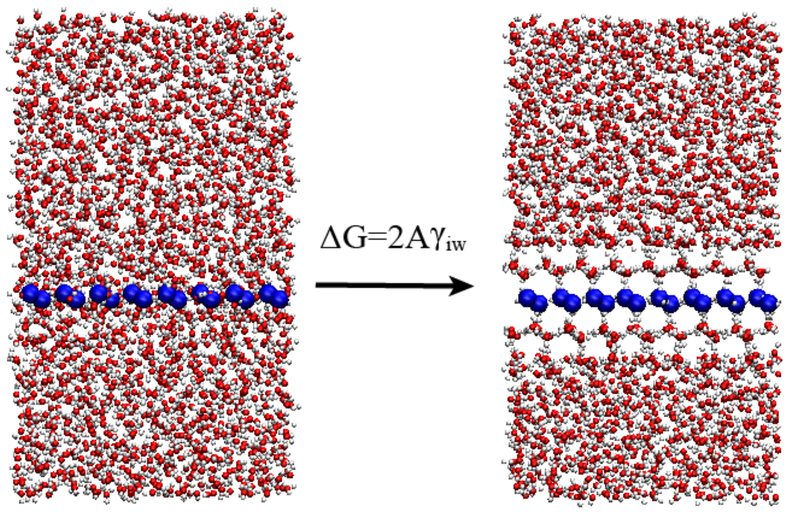


**Figure S14** Snapshots of a TIP6P liquid-ice coexistence configuration at 1 bar pressure and 289 K. The mold of potential energy wells is represented by the blue spheres. Its interaction with water molecules is switched off in left and on in right panel.

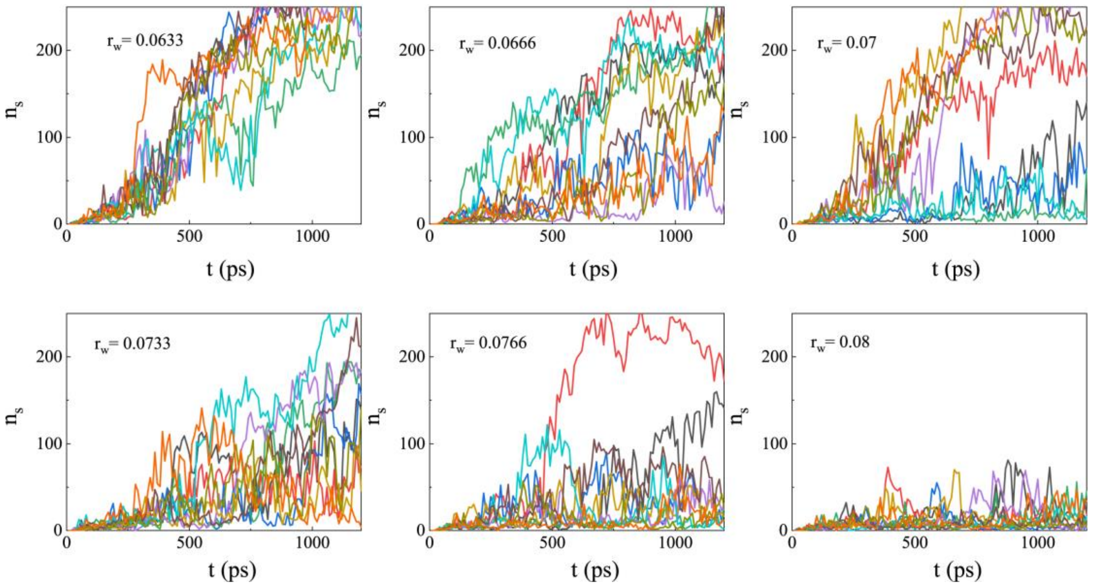


**Figure S15** Number of particles in the largest crystal cluster $\mathbf{n_s}$ versus time for several trajectories and different well radii (as indicated in the legend in nm) for the basal plane of the TIP6P model. All simulations are performed at coexistence conditions (1 bar, 289 K). In all cases $\boldsymbol{\varepsilon = \varepsilon_m = 8\,k_BT}$. If $\mathbf{r_w} \leq \mathbf{r_w^0}$, an ice slab should grow in all trajectories with no induction period given that there is no free energy barrier for the formation of the crystal. On the contrary, if $\mathbf{r_w} \geq \mathbf{r_w^0}$, a barrier must be overcome, which is reflected in some trajectories having an induction period before the slab grows or even in no slab formation at all if $\mathbf{r_w}$ is too large.Therefore, we find $\mathbf{r_w^0}$ = 0.06495 $\pm$ 0.005 nm.

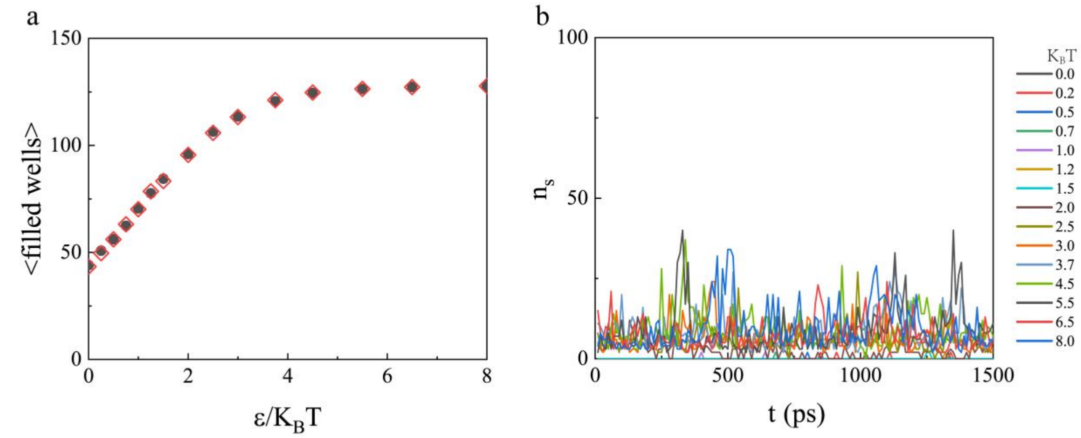


**Figure S16 a** Average number of filled wells versus well depth ($\boldsymbol{\varepsilon/k_BT}$) for the basal plane of the TIP6P model. The radius of the mold wells is 0.08 nm. All simulations are performed at 1 bar and 289 K. Black circles (empty diamonds) correspond to simulations starting from an equilibrated configuration with the mold switched off (on). **b** $\mathbf{n_s}$ versus time for all integration points shown in a. Indicated in the legend is the well depth in $\mathbf{k_BT}$.

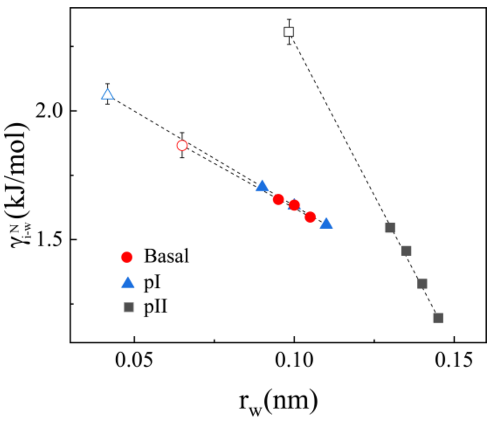


**Figure S17** Surface free energy for different values of the well radius and crystal orientations of ice Ih, as indicated by the filled symbols in the legend (statistical errors have the size of the symbols). The dashed lines represent linear fits to the filled symbols, while the empty symbols indicate the extrapolation of these linear fits to the optimal well radius.

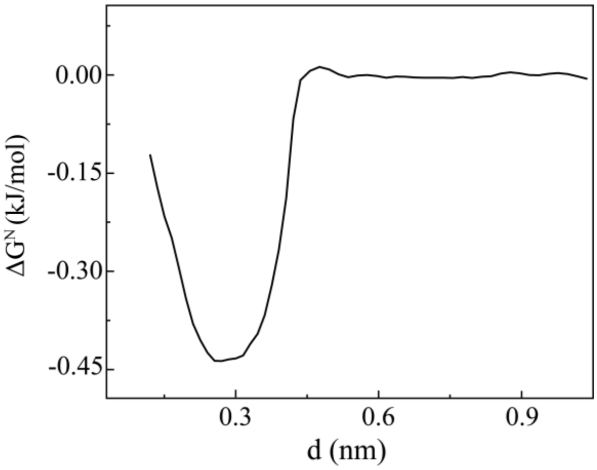


**Figure S18** Binding free energy of the TmAFP protein to ice as function of the distance between the center of mass of the protein and the ice core surface.

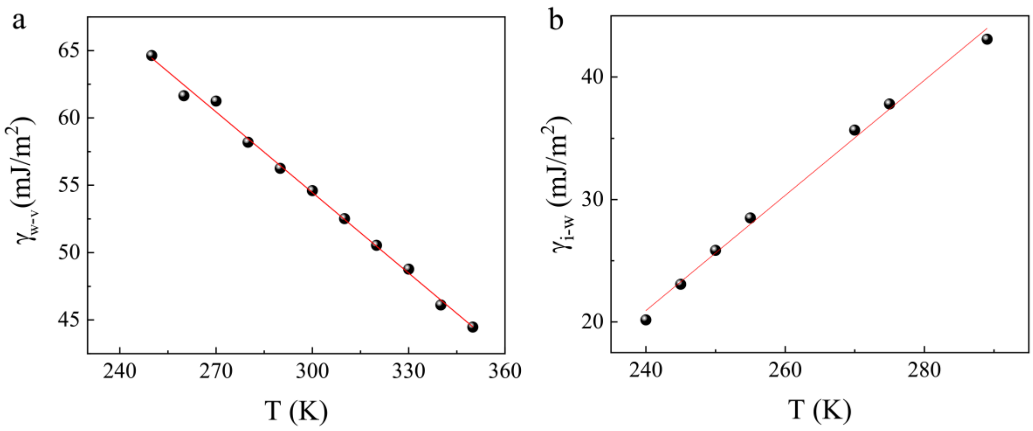


**Figure S19** Temperature dependence of surface tension of the water-vapor surface **a** and the ice-water interface **b**.

## Supporting Movies

**Movie S1 (separate file).** Simulated ice nucleation process on the IBS of TmAFP with the protein's main chain immobilized. To simplify the model, only the main chain, along with the methyl and hydroxyl groups on the IBS are displayed for clarity.

**Movie S2 (separate file).** Simulated dynamic behavior of confined water within the threonine groove on the IBS of native TmAFP protein.

**Movie S3 (separate file).** Simulated dynamic behavior of confined water within the serine groove on the IBS of Mu1 protein. Water molecules in the inverted groove diffuse into the bulk water, after which the molecules in the groove are re-labeled to track the movement behavior of those previously occupied the inverted groove.

## Supporting References


1. Hudait A, Moberg DR, Qiu Y, Odendahl N, Paesani F, Molinero V. Preordering of water is not needed for ice recognition by hyperactive antifreeze proteins. *Proc. Natl. Acad. Sci. U. S. A.* **2018**, 115, 8266-8271.

2. Mickel W, Kapfer SC, Schröder-Turk GE, Mecke K. Shortcomings of the bond orientational order parameters for the analysis of disordered particulate matter. *J. Chem. Phys.* **2013**, 138, 4.

3. Reinhardt A, Doye JPK, Noya EG, Vega C. Local order parameters for use in driving homogeneous ice nucleation with all-atom models of water. *J. Chem. Phys.* **2012**, 137, 194504.

4. Zhu X, Vandamme M, Jiang Z, Brochard L. Molecular simulation of the confined crystallization of ice in cement nanopore. *J. Chem. Phys.* **2023**, 159, 154704.

5. Mackerell Jr AD, Feig M, Brooks Iii CL. Extending the treatment of backbone energetics in protein force fields: Limitations of gas-phase quantum mechanics in reproducing protein conformational distributions in molecular dynamics simulations. *J .Compute. Chem.* **2004**, 25, 1400-1415.

6. Nguyen AH, Molinero V. Identification of Clathrate Hydrates, Hexagonal Ice, Cubic Ice, and Liquid Water in Simulations: the CHILL+ Algorithm. *J. Phys. Chem. B* **2015**, 119, 9369-9376.

7. Lupi L, Peters B, Molinero V. Pre-ordering of interfacial water in the pathway of heterogeneous ice nucleation does not lead to a two-step crystallization mechanism. *J. Chem. Phys.* **2016**, 145, 211910.

8. Wang C, *et al.* Stable Liquid Water Droplet on a Water Monolayer Formed at Room Temperature on Ionic Model Substrates. *Phys. Rev. Lett.* **2009**, 103, 137801.

9. Nutt DR, Smith JC. Dual Function of the Hydration Layer around an Antifreeze Protein Revealed by Atomistic Molecular Dynamics Simulations. *J. Am. Chem. Soc.* **2008**, 130, 13066-13073.

10. Gregory JK, Clary DC, Liu K, Brown MG, Saykally RJ. The Water Dipole Moment in Water Clusters. *Science* **1997**, 275, 814-817.

11. Li S, Chen Y, Zhao J, Wang C, Wei N. Atomic structure causing an obvious difference in thermal conductance at the Pd–H2O interface: a molecular dynamics simulation. *Nanoscale* **2020**, 12, 17870-17879.

12. Li L, *et al.* Resolving Temperature-Dependent Hydrate Nucleation Pathway: The Role of "Transition Layer". *J. Am. Chem. Soc.* **2023**, 145, 24166-24174.

13. Nada H, van der Eerden JPJM. An intermolecular potential model for the simulation of ice and water near the melting point: A six-site model of H2O. *The J. Chem. Phys.* **2003**, 118, 7401-7413.

14. Espinosa JR, Vega C, Sanz E. Ice–Water Interfacial Free Energy for the TIP4P, TIP4P/2005, TIP4P/Ice, and mW Models As Obtained from the Mold Integration Technique. *J. Phys. Chem. C* **2016**, 120, 8068-8075.

15. Abascal JLF, Fernández RG, Vega C, Carignano MA. The melting temperature of the six site potential model of water. *J. Chem. Phys.* **2006**, 125, 16.

16. Qiu Y, Hudait A, Molinero V. How Size and Aggregation of Ice-Binding Proteins Control Their Ice Nucleation Efficiency. *J. Am. Chem. Soc.* **2019**, 141, 7439-7452.

17. Qiu Y, Lupi L, Molinero V. Is Water at the Graphite Interface Vapor-like or Ice-like? *J. Phys. Chem. B* **2018**, 122, 3626-3634.
